# An Adaptive Transfer Learning Framework for Multimodal Autism Spectrum Disorder Diagnosis

**DOI:** 10.3390/life15101524

**Published:** 2025-09-26

**Authors:** Wajeeha Malik, Muhammad Abuzar Fahiem, Jawad Khan, Younhyun Jung, Fahad Alturise

**Affiliations:** 1 Department of Computer Science, Lahore College for Women University, Lahore 54500, Pakistan; wajeeha.malik@lcwu.edu.pk (W.M.); abuzar@lcwu.edu.pk (M.A.F.); 2 School of Computing, Gachon University, Seongnam 13120, Republic of Korea; jkhanbk1@gachon.ac.kr; 3 Department of Cybersecurity, College of Computer, Qassim University, Buraydah 51452, Saudi Arabia

**Keywords:** autism spectrum disorder, multimodal classification, feature engineering, machine learning, multi-layer perceptron, deep learning, transfer learning, fusion model

## Abstract

Autism Spectrum Disorder (ASD) is a complex neurodevelopmental condition with diverse behavioral, genetic, and structural characteristics. Due to its heterogeneous nature, early diagnosis of ASD is challenging, and conventional unimodal approaches often fail to capture cross-modal dependencies. To address this, this study introduces an adaptive multimodal fusion framework that integrates behavioral, genetic, and structural MRI (sMRI) data, addressing the limitations of unimodal approaches. Each modality undergoes a dedicated preprocessing and feature optimization phase. For behavioral data, an ensemble of classifiers using a stacking technique and attention mechanism is applied for feature extraction, achieving an accuracy of 95.5%. The genetic data is analyzed using Gradient Boosting, which attained a classification accuracy of 86.6%. For the sMRI data, a Hybrid Convolutional Neural Network–Graph Neural Network (Hybrid-CNN-GNN) architecture is proposed, demonstrating a strong performance with an accuracy of 96.32%, surpassing existing methods. To unify these modalities, fused using an adaptive late fusion strategy implemented with a Multilayer Perceptron (MLP), where adaptive weighting adjusts each modality’s contribution based on validation performance. The integrated framework addresses the limitations of unimodal approaches by creating a unified diagnostic model. The transfer learning framework achieves superior diagnostic accuracy (98.7%) compared to unimodal baselines, demonstrating strong generalization across heterogeneous datasets and offering a promising step toward reliable, multimodal ASD diagnosis.

## 1. Introduction

Autism Spectrum Disorder (ASD) is a neurodevelopmental disorder that significantly affects individuals’ perception of the world, influencing how they interpret, engage with, and respond to their surroundings [1]. The disorder usually emerges early in life and persists throughout an individual’s developmental trajectory, with varying severity levels. The symptoms of ASD vary widely among individuals, making it challenging to provide a one-size-fits-all description of the disorder.

ASD presents a spectrum of conditions that share common characteristics including challenges in non-verbal communication, such as difficulties in interpreting and using gestures, facial expressions, and body language. Individuals with ASD often display focused and intense engagement in limited or repetitive activities that reflect specific hobbies or routines. ASD presents a heterogeneous range of symptoms, including difficulties in social communication, repetitive behaviors, and sensory sensitivities, which vary widely across individuals [2]. This heterogeneity complicates both clinical assessment and automated diagnosis.

According to the World Health Organization (WHO), ASD affects approximately 100 children worldwide [3]. with higher prevalence in males [4]. Diagnosis typically occurs around age five [5], but earlier detection is critical since timely intervention can significantly improve developmental outcomes [6,7].

Although the etiology of ASD remains unclear, both genetic and environmental factors are believed to play a role in its prevalence [8]. This diagnostic gap has motivated researchers to explore computational intelligence techniques for objective and scalable ASD detection [9,10,11]. Multiple machine learning models such as support vector machine (SVM), XG Boost, and random forests have also been implemented on behavioral data and genetic data and have demonstrated potential results for the classification of ASD based on extracted features [12,13]. However, most existing studies are unimodal, focusing on either behavioral, genetic, or neuroimaging data in isolation, and thus fail to capture inter-modal dependencies [14]. Furthermore, variability in data sources reduces the generalizability of these models [15]. To overcome these challenges, this study presents an adaptive multimodal fusion framework that integrates behavioral, genetic, and sMRI data. Unlike unimodal approaches, the framework explicitly models cross-modal relationships, enhancing diagnostic accuracy and robustness.

The principal contributions of this study are as follows.

1. Development of an adaptive multimodal fusion framework that integrates behavioral, genetic, and sMRI modalities, improving generalization compared to unimodal baselines.
2. Introduction of a hybrid CNN–GNN architecture for sMRI analysis, capturing both local spatial features and brain connectivity patterns.
3. Comprehensive evaluation across heterogeneous datasets, demonstrating superior accuracy and robustness relative to state-of-the-art ASD diagnostic methods.

The following section reviews related work on unimodal and multimodal approaches for ASD diagnosis, highlighting the gaps this study addresses.

## 2. Literature Review

For the early diagnosis of ASD, machine learning (ML) approaches have recently become quite well-known. Numerous studies have tried to maximize the speed of and exactness of ASD identification using machine learning techniques. One of the research presented a Rules Machine Learning (RML) technique to assess ASD qualities [16]. This rule-based method not only offered valuable insights into classification decisions but significantly improved classifier performance also, surpassing conventional methods like Boosting and decision trees (DT).

However, the reliance on handcrafted rules limits scalability to diverse datasets and larger population cohorts. The author conducted a study on attribute selection in ASD screening, crucial for classification accuracy [17]. Various methods like correlation feature set, information gain, fast correlated-based filter, Chi-square, and Gini index were used to identify most important autistic traits in a dataset with over 1000 toddler observations. K-Nearest Neighbor (KNN), ID3, and AdaBoost were employed to build models based on different datasets selected using attribute selection methods. Main findings revealed that these attribute selection methods could enhance the accuracy of the classifier and pre diagnosis process. In 2021, researchers constructed ML based early-detection model for ASD in children [18]. Five classification models including Random Forest (RF), Naïve Bayes, Logistic Regression, KNN, and Support Vector Machine (SVM) were employed on the toddler dataset [19] consisting of 1054 instances and 18 features. The experimental results revealed that the Logistic Regression had the best F1 score (0.98) and precision (97.15%).

Recent research employed several feature selection techniques on ML models to identify early behavioral indicators for ASD in toddlers [20]. For early screening, the researchers highlighted the significance of cognitive features associated with social interaction, communication, and stereotyped behaviors. Logistic regression and Bayesian network models demonstrated consistent predictive accuracy. There is a study focusing on predicting ASD risk across different age groups [21]. A range of ML and deep learning (DL) algorithms, including Naive Bayes, Logistic Regression, Convolutional Neural Networks (CNN), SVM, KNN and Neural Networks were employed on datasets representing children, adults, and adolescents. The results highlighted the superior performance of CNN in detecting ASD, with accuracy rates of 99.53% for adults, 98.30% for children, and 96.88% for adolescents.

A study conducted in 2023, employed similar ML models for predicting ASD traits across individuals of varying ages [22]. Four publicly available datasets of 1054 toddlers, 292 children, 104 adolescents, and 704 adults were used. These datasets had 21 traits except for toddlers having 19 traits. The finding revealed that LR, RF and DT classifiers achieved the highest prediction accuracy of approximately 85–90% for all datasets. In a related research [23], four same datasets (toddlers-adults) were used for automating the ASD diagnosis process. This study employed five feature selection methods to reduce features and 27 classification algorithms. The results highlighted the superior performance multilayer perceptron (MLP) classifier showing achieving 91 accuracy and the effectiveness of the Relief F feature selection technique for ranking important traits. The main limitation was the use of small datasets, impacting the generalizability of the findings.

Another study [24] proposed a ML model to diagnose ASD accurately across different age levels. Feature selection techniques, e.g., RIPPPER, Correlation based, and Boruta algorithm were used for generating feature subsets. SVM outperformed other classifiers with 97.82% accuracy for toddler, 99.61% accuracy for children, 95.87% accuracy for adolescent, and 96.82% accuracy for the adult subset. Further, the interpretability of the model was improved by ranking features using SHAP method. Another study used some other types of screening tests called Q-CHAT-10 and AQ-10 questionnaires for toddlers, children, adolescents, and adults. There were different questions in the questionnaire about one’s behavior. An app developed by the Nelson Marlborough Institute of Technology was used to calculate screening score ranges from 1 to 10 for the participants according to their answers to the questions. A score higher than 6 meant that the participant had ASD. Different machine learning models were also used to predict the screening score. For these models, various feature selection techniques were used from which Correlation-based Feature Selection (CFS) and Boruta algorithm gave the best results. The highest accuracy was achieved by an SVM model.

The researchers in 2023 proposed five feature selection techniques for ranking most important traits to diagnose ASD in toddlers, children, adolescents, and adults [25]. From 27 benchmark classifiers, MLP achieved 100% accuracy with minimal features across all age groups. Relief F approach was recommended for selecting less features with competitive performance. An unsupervised ML method based on a two-phase system was proposed for assessing the predictive accuracy of ASD screening [26]. The first phase (pre-diagnostic) used self-organizing map (SOM) to cluster the input. This phase refined the dataset and achieved 85% accuracy. During the next phase (classification), refined data was used with Naïve Bayes and RF algorithms. The RF classifier outperformed the NB classifier, showing high performance with 97% recall, 96% accuracy, and 96% precision.

A framework for evaluating eight ML techniques and four feature scaling strategies was introduced for detecting ASD [27]. The study was conducted on four datasets (toddler-adult). AdaBoost, when scaled with normalizer, achieved 99.25% accuracy for toddlers and 97.95% for children. Linear Discriminant Analysis with Quantile Transformer yielded the best results for Adolescents (97.12%), and adults (99.03%). Feature importance analysis using feature selection techniques were also the contribution of this study.

Genetic testing is another modality that medical professionals use for detecting ASD.

While behavioral-based machine learning approaches demonstrate strong predictive accuracy, they are constrained by subjective assessments and dataset size. To address biological underpinnings of ASD, researchers have turned toward genetic analysis as another complementary modality [28].

De novo mutations in genes are used to diagnose ASD. Find the precise collection of genes impacted by the mutations. Machine learning techniques including boosted trees, random forests, support vector machines, and logistic regression were applied by them. Inputs were provided of various genetic variants including network traits, spatiotemporal gene expression patterns, and gene-level constraint measures. According to the results, MYCBP2, CAND1, TCF20, HERC1, and NBEA are the genes most affected by ASD. This shows the role of genetic data in detection of ASD [29]. Nonetheless, genetic-only models often face challenges of interpretability and limited predictive power due to polygenic and heterogeneous contributions to ASD risk [30].

In another study, they analyzed common genetic variants to predict ASD using a deep learning model. They obtained the data for this study from Simons Simplex Collection (SSC). The genetic variants were ranked according to their importance using chi-square tests and the top 100 variants were selected to form a feature vector. This feature vector was fed to a Convolutional Neural Network (CNN). They achieved an AUC of 0.955 and an accuracy of 88.6%.

A study was conducted that utilized a deep neural network (DNN) classifier for performing the classification of ASD/TC using MRI images. After constructing the individual brain network and extracting the morphological features of each subject, the connectivity between each pair of ROIs was extracted. Based on the F score, these connectivity features were categorized in descending order and the 3000 features on the top were selected. After the selection, ASD/TC classification was performed via a DNN classifier. Evaluating their method on T1-weighted MRI data from the ABIDE I dataset with ten-fold cross-validation, the proposed method achieved impressive results: a classification accuracy of 90.39% [31].

Despite strong results, deep MRI-based models often require large sample sizes and advanced preprocessing pipelines, which may limit their reproducibility across clinical settings. This study introduces an innovative approach by utilizing fMRI data from the ABIDE database together with deep learning techniques and brain frequencies to distinguish between 84 school-aged children with ASD and typically developing counterparts. This model suggested preprocessing of the data followed by decomposing it into 30 independent components for every child. Subsequently, the selected ICs were entered into a stacked auto-encoder (SAE) to reduce dimensionality and perform feature subtraction. In the last step, the school-aged children suffering from autism were separated from the Typical Development (TD) school-aged children by using the SoftMax classifier. The results demonstrated an impressive average accuracy of 87.21% [32].

The use of sMRI for the detection of ASD introduced a multivariate classification approach that used sMRI data for classifying autism. It indicated whether the direction of the gross morphological patterns of the brain of each individual aligned with the autism cases or typically developing controls. The morphometric analysis found that there were substantial differences identified within limbic areas and subcortical gray matter. However, no differences were identified in the total volume of the brain. In an experimental study involving individuals with and without ASD, the classifier achieved a maximum accuracy of 78.9% following cross-validation that demonstrated a higher accuracy performance [33].

A method was proposed that utilized both functional and structural MRI information for classifying ASD patients versus control subjects. It used volume-based comparisons of grey matter regions across cortical parcels and functional connectivity patterns across brain regions as features for distinct processing pipelines. The classification framework combined stacked autoencoders trained without supervision and multilayer perceptron’s trained under supervision. Classification precision of 85.06 ± 3.52% was obtained by utilizing the classifiers together. By integrating evidence from both functional and structural MRI sources, the study demonstrates the significant performance gains compared to the individual modalities. The study stresses how crucial multimodal analysis is in improving ASD diagnosis [34].

Another study addressed the diagnostic challenges associated with the increasing number of ASDs cases in Malaysia by employing neuroimaging fMRI data, the research applied deep learning techniques, specifically variants of CNNs, to extract the robustness of features from the neuroimages in fMRI, and on the basis of those features, it detects the presence of ASD in patients. For the classification of neural patterns, the performance of pre-processed images was interpreted. Utilizing the ABIDE dataset, the study evaluated the performance of CNN models like VGG-16 and ResNet-50 and achieved accuracies of 63.4% and 87.0%, respectively [35]. Sha et al. (2025) [36] presented a fusion model combining facial image processing and demographic features via SBiLSTM and attention-based pooling, achieving an accuracy of 99.2%. Although impressive, their reliance on visual features limits biological interpretability compared to models that incorporate MRI or genomics. Ref. [37] proposed a robot-assisted, sensor-driven multimodal framework focusing on real-time body dynamics (eye gaze, head movement, and skeleton data) using ensemble learning. Although it showed high accuracy (97.36%), it remains dependent on specific environmental setups and real-time robotic interfaces, limiting generalization.

Finally, Gao, L. et al. (2025) [38] used unsupervised similarity network fusion on multimodal MRI to subtype ASD populations but did not provide predictive performance metrics, focusing more on clustering and subgroup discovery than diagnostic classification. LSTVision [39] and MCBERT [40] utilize advanced neural architectures such as LSTM, Transformers, and BERT to model the temporal or textual aspects of the data, showing promising results on public datasets such as ABIDE [41], for instance, MCBERT achieved a classification accuracy of 93.4% by combining a CNN with textual embeddings from BERT. However, these models are limited in scope by focusing primarily on fMRI and text features and lacking the integration of core behavioral data or genomics. Gao et al.’s [42] hierarchical multimodal deep feature integration model introduced a structured multi-scale extraction strategy for rs-fMRI, yet it excludes behavioral or molecular-level data, potentially missing important phenotypic and genetic signals. Song et al. (2025) [43] proposed a dual transformer model combined with Graph Convolutional Networks (GCNs) for processing sMRI and fMRI data, attaining a maximum accuracy of 79.47%.

Despite their novelty in incorporating graph-based structures, their relatively low performance highlights the challenges of neuroimaging-only methods.

In existing literature, most ASD diagnostic studies rely on unimodal datasets, each representing behavioral, genetic, or neuroimaging information in isolation. While this modality-specific approach enables the identification of age- or domain-specific ASD traits, it suffers from critical limitations, including data scarcity, reduced diagnostic robustness, and limited generalizability across populations. Moreover, it cannot provide a holistic understanding of ASD characteristics across the human lifespan. These limitations have motivated increasing interest in multimodal fusion techniques, which seek to combine complementary strengths of behavioral assessments, genetic profiles and neuroimaging scans to improve both generalization and robustness in timely ASD diagnosis.

## 3. Proposed Methodology

The proposed Multimodal framework is a fusion strategy that integrates to improve diagnostic accuracy, behavioral, genetic, and structural MRI (sMRI) data from fusion-based techniques have been shown to outrank single-modal models in related medical imaging.

Each modality is processed independently using specialized model ensemble learning classifiers are applied to behavioral and genetic datasets, while a deep learning-based architecture is used for the sMRI modality. This choice reflects the nature of the input data: behavioral and genetic features are tabular and benefit from ensemble-based classifiers, whereas sMRI data contains high-dimensional spatial information, making deep learning architectures more appropriate.

The predicted probabilities from these individual classifiers are then combined at the decision level through a late fusion mechanism wherein a custom designed Multi- Layer Perceptron (MLP) classifier is employed to perform the final diagnosis of ASD, enabling the framework to capture complementary patterns across data types. This fusion-driven architecture not only improves overall diagnostic performance but also provides a robust and scalable solution for early ASD detection across different age groups and clinical environments. Following this, several machine learning algorithms have been developed and refined via strict hyperparameter optimization methods [44]. The results obtained from the proposed multimodal were comparatively evaluated using performance metrics and validation methods enhance generalization. The proposed methodology is described in the following subsections. Behavioral datasets were obtained from [19,45,46] and genetic datasets were obtained from [47], and preprocessed. Ensemble classifiers were chosen based on prior studies showing improved robustness on tabular neurobehavioral and genomics data.

### 3.1. Multimodal Fusion Modeling

This section outlines the methodology used to integrate three pretrained models trained on distinct data types (MRI, behavioral, and genetic) into a unified multimodal fusion architecture for autism classification [48,49]. The approach aims to make use of complementary features from each modality to enhance classification performance.

#### 3.1.1. Overview of the Fusion Strategy

The proposed Fusion Model combines latent representations learned independently from three previously trained deep neural networks:

1. MRI model for structural imaging features (128-dim output)
2. Behavioral model for neuropsychological/behavioral metrics (32-dim output)
3. Genetic model for genomics-based signatures (64-dim output)

These three streams are concatenated to form a comprehensive 224-dimensional fused vector that is passed through a multi-layer feedforward classifier [50,51,52]. Prior to concatenation, feature vectors from all modalities were normalized to ensure comparable scales across heterogeneous inputs.

**Figure 1:**
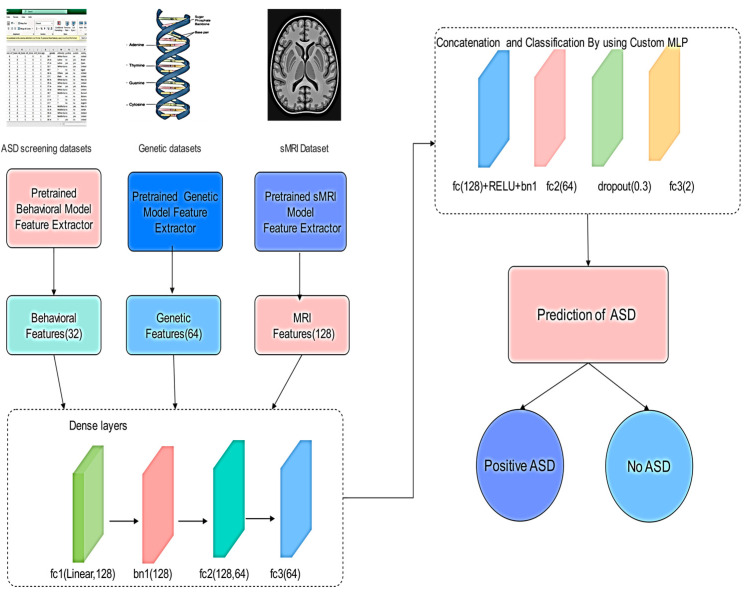
Multimodal Fusion Architecture Diagram for ASD Classification.

The MLP is used for late fusion as it provides sufficient capacity to model interactions across heterogeneous modalities while allowing regularization techniques (dropout and batch normalization) to prevent overfitting. This architecture is computationally efficient compared to more complex models and captures complementary patterns across data types effectively. The late fusion MLP adaptively reweights modality contributions during training, effectively adjusting the influence of each unimodal embedding based on its predictive utility (Figure 1).

fc1 (Fully Connected Layer 1): Projects the concatenated multimodal feature vector into a higher-level feature space, enabling interaction between behavioral, genetic, and sMRI features.

bn1 (Batch Normalization): Normalizes the outputs of fc1 to stabilize training, reduce internal covariate shift, and improve convergence speed.

fc2 (Fully Connected Layer 2): Further compresses and refines the joint feature representation, encouraging the network to learn more compact and discriminative embeddings.

fc3 (Fully Connected Layer 3/Output Layer): Produces the final prediction logits for ASD classification.

The proposed methodology illustrates the multimodal fusion framework for ASD classification. As mentioned before, the system integrates three pre-trained unimodal feature extractors to process behavioral scores, genetic profiles, and structural MRI data. Each modality yields a low-dimensional embedding (32, 64, and 128 features), which are concatenated into a 224-dimensional vector. This vector was then passed through a custom MLP feedforward neural network comprising fully connected layers, batch normalization, ReLU activation, and dropout for regularization. The final output is a binary prediction of the autism status. This architecture effectively captures both modality-specific patterns and cross-modality interactions, leveraging the complementary nature of neuroimaging, behavioral, and genomic data.

(1) $$x_{mri}\in R^{128}\;x_{beh}\in R^{32}\;x_{gen}\in R^{64}\;\mathrm{The}\;\mathrm{fused}\;\mathrm{vector}\;\mathrm{is}:\;x_{fused}=\left[x_{mri},x_{beh},x_{gen}\right]\in R^{224}$$

Equation (1) corresponds to the fusion step right and it concatenates the feature vectors.

Equation (2) “fc1(128 + ReLU + bn1)”, applies Batch Normalization and ReLU Activation. This vector is processed as follows:

(2) $$h_{1}=ReLU\left(BN\left(W_{1}x_{fused}+b_{1}\right)\right)\left(BatchNorm+DenseLayer1\right)$$

Equation (3) matches the second layer “fc2(64)” and applies ReLU with it. The vector is processed as below:

(3) $$h_{2}=ReLU\left(W_{2}h_{1}+b_{2}\right)\left(DenseLayer2\right)$$

Equation (4) applies dropout to reduce the overfitting with probability of 0.3. The vector is shown as:

(4) $$h_{3}=Dropout\left(h_{2},p=0.3\right)$$

Equation (5) corresponds to “fc3(2)” and leads to prediction of ASD, its vector is written in Equation (5) below:

(5) $$\hat{y}=W_{3}h_{2}+b_{3}\left(Output\;Layer\right)$$

where $\hat{y}\in R^{2}$ is the output logits for binary Classification (Autism vs. Control)

(6) $$L=-\sum_{i=1}^{2}y_{i}log(\hat{y_{i}})$$

The overall optimization objective is defined by the cross-entropy loss function in Equation (6), where y denotes the ground truth label and $\hat{y}$ represents the predicted probability distribution:

#### 3.1.2. Training Procedure

To train the multimodal fusion model, a synthetic dataset comprising 500 samples was generated. The synthetic dataset was created to simulate realistic distributions of multimodal ASD data by sampling feature vectors from Gaussian distributions parameterized using statistics derived from publicly available ASD datasets. This approach ensured that the synthetic data retained representative modality-specific characteristics while enabling controlled evaluation of the fusion model. The dataset was split into training, testing, and validation subsets. Specifically, 70% of the data (350 samples) was allocated for training, 20% (100 samples) for testing, and 10% (50 samples) of the training set was reserved for validation purposes. Each data sample consisted of a tuple of three modality vectors along with a binary label.

The Adam optimizer with a learning rate of 0.001 was used in the training process. Over 50 epochs, the model was trained with a batch size of 32. The loss function used was Cross-Entropy Loss, which is suitable for binary classification tasks. Importantly, during training, only the fusion layers of the model were updated through backpropagation, while the modality-specific sub-models remained frozen, preserving their pre-trained weights.

To ensure the synthetic dataset captures realistic inter-modal relationships, statistical correlations observed in publicly available ASD datasets between behavioral, genetic, and MRI features were preserved. Specifically, covariance structures and modality-specific distributions were maintained during sampling, enabling the model to learn realistic cross-modal patterns despite the controlled synthetic data setting. Due to the scarcity of publicly available datasets containing all three modalities, synthetic data was employed to simulate multimodal integration while preserving realistic inter-modal correlations.

Hyperparameters for the fusion model, including learning rate, number of layers, dropout rate, and batch size, were selected through a grid search using the validation subset to optimize the F1-score. Early stopping based on validation loss was also employed to prevent overfitting.

The key training parameters are summarized in Table 1 below:

#### 3.1.3. Model Evaluation

After the training phase, the model’s performance was assessed through several evaluation metrics. Accuracy was measured for both the training and validation sets to monitor learning progress. Additionally, the loss values for both sets were tracked across epochs to identify underfitting or overfitting trends.

**Figure 2:**
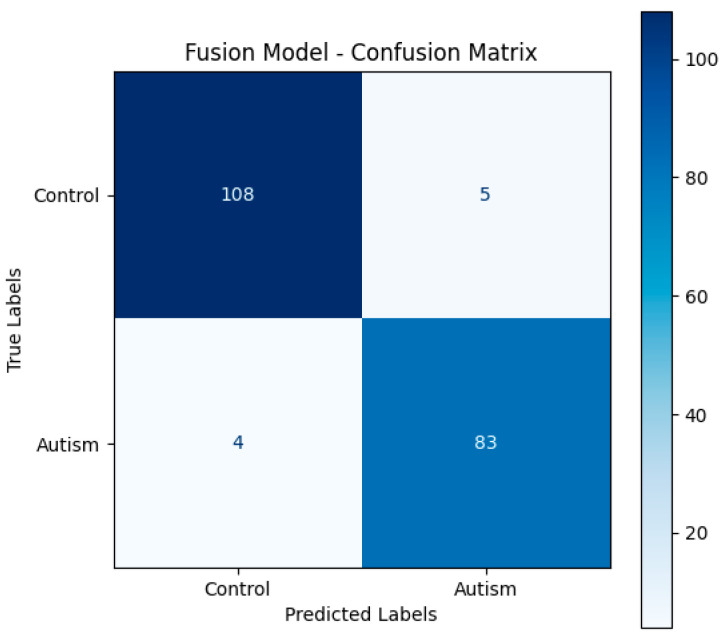
Fusion Model Confusion Matrix.

To better understand the model’s classifying performance across both classes, the evaluation also included a confusion matrix generated on the test set. Moreover F1-score and Area Under the Receiver Operating Characteristic Curve (AUC) were also calculated for accuracy, therefore offering a more trustworthy evaluation under possible class imbalance. Hyperparameters were tuned and learning curves were monitored using the validation subset to guarantee that the stated accuracy, F1-score, and AUC results reflect generally acceptable performance rather than Figure 2 shows the Fusion Model Confusion Matrix.

#### 3.1.4. Summary of Fusion Model Parameters

Table 2 below shows that the fusion model integrates features from three pretrained unimodal networks (MRI, Behavioral, Genetic), producing a fused input vector of size 224. This vector is processed using a 3-layer fully connected classifier with ReLU activation. Regularization techniques include batch normalization (applied after the first layer) and dropout (rate = 0.3). The model was trained for 50 epochs using the Adam optimizer and cross-entropy loss, with an 80/20 training-to-testing data split. The following section presents the experimental results of the proposed framework, including unimodal baselines and multimodal.

### 3.2. Behavioral Data Analysis

**Figure 3:**
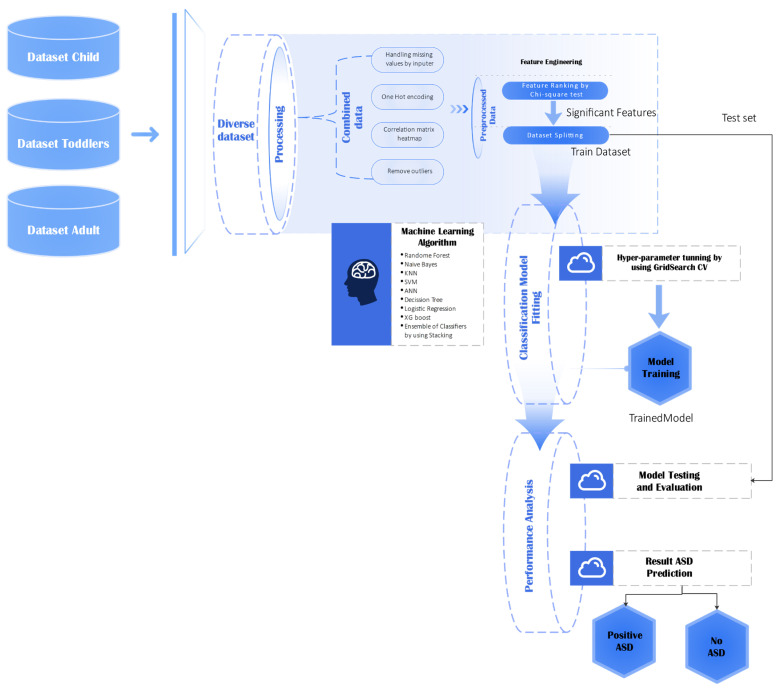
Workflow Diagram for Behavioral Data Analysis.

This section describes the method applied to analyze behavioral data across various age groups (child, toddler, and adult) to predict autism spectrum disorder (ASD). The entire machine learning pipeline, from raw data importation to the end model assessment, is depicted in Figure 3. This process guarantees the integrity, scalability, and reproducibility of analysis.

This diagram illustrates the end-to-end pipeline of dataset preparation, preprocessing, feature engineering, classifier training, and testing for the prediction of ASD.

The dataset was first subjected to a series of preprocessing steps to ensure compatibility with the learning algorithms and to maximize model performance [44]. Categorical features were converted into numeric form using both label encoding and one-hot encoding, enabling the models to process discrete variables efficiently. The dataset was stratified and split into training (70%), testing (20%), and validation (10%) subsets to maintain class balance. Numerical features were standardized using the StandardScaler, fitted on the training data and applied to all subsets to prevent data leakage. The dataset underwent several preprocessing steps to prepare the model and achieve top-notch performance:

For baseline performance evaluation, a Decision Tree classifier was implemented due to its interpretability and computational simplicity. Hyperparameter tuning was conducted via GridSearchCV using a 5-fold cross-validation strategy, optimizing over the criterion (Gini, entropy) and maximum tree depth values (3, 5, 7, and 10) [53,54,55]. The best-performing model was selected based on cross-validation accuracy and was subsequently evaluated on the test set using precision, recall, F1-score, and overall accuracy as performance metrics [56]. These preprocessing steps ensure consistent feature scales across samples, handle missing values, and maintain class balance, which is essential for subsequent multimodal integration and accurate fusion-based classification.

To build upon the baseline, an Extreme Gradient Boosting (XGBoost) classifier was employed, initialized with random_state = 42, use_label_encoder = False, and eval_metric = ‘logloss’ [57]. A grid search was performed over the number of estimators (50, 100, 200), maximum tree depth (3, 6, 9), learning rate (0.01, 0.1, 0.2), subsample ratios (0.7, 0.8, 0.9), and column subsample ratios (0.7, 0.8, 0.9) [58]. Parallel processing (n_jobs = −1) was applied to improve training efficiency [59]. To speed up the process, all computations used parallel processing (n_jobs = −1). The evaluation followed the same metrics used for the Decision Tree.

In parallel, an Artificial Neural Network (ANN) was developed using Keras with a TensorFlow backend to capture complex nonlinear interactions within the data [60,61]. The network consisted of an input layer sized to the number of features, two hidden layers with 64 units each and ReLU activation, and an output layer with a single sigmoid-activated neuron for binary classification. The model was trained over 100 epochs using the Adam optimizer with a learning rate of 0.001 and binary cross-entropy as the loss function. A 5-fold cross-validation approach was used for model selection, leveraging classification accuracy as the primary metric [62,63]. Thresholding output probabilities at 0.5 yielded the model’s ultimate predictions; performance was evaluated using the same battery of criteria of accuracy, precision, recall, and F1-score. To ensure robust performance across heterogeneous behavioral datasets, all base learners were evaluated using 5-fold cross-validation, and the stacking ensemble combined their out-of-fold predictions to avoid overfitting. This approach leverages complementary strengths of tree-based and neural methods, improving generalization while preserving interpretability The hyperparameters used for model training are summarized below in Table 3.

#### Stacking Ensemble Classifier

To harness the complementary strengths of different learning algorithms, a stacking ensemble classifier was developed. This ensemble combined three diverse base learners: a tuned Random Forest model, the best-performing XGBoost classifier, and an ANN implemented using the MLPClassifier with two hidden layers [64,65].

The outputs of these base learners were used as input features for a meta-learner, which was a Logistic Regression classifier. This meta-model was trained on the out-of-fold predictions of the base learners to prevent data leakage and overfitting [66]. While stacking ensembles are established techniques, their application here is tailored to maximize complementary information from behavioral data, forming an optimized feature representation for later multimodal fusion. The final prediction of the ensemble model is given by:

(7) $$\hat{y}=\sigma \left(w_{1}{\hat{y}}_{rf}+w_{2}{\hat{y}}_{xgb}+w_{3}{\hat{y}}_{ann}+b\right)$$

where σ is the sigmoid function, ${\hat{y}}_{model}$ is the base model prediction, and $w_{i}$ are the weights learned by logistic regression.

The stacking model was evaluated on the test set using the same suite of performance metrics as applied to the individual classifiers [67]. Visualization tools were also employed to further interpret the model’s effectiveness. The optimized stacking ensemble achieved an overall accuracy of 95.5%, consistent with the abstract, confirming reliable behavioral feature extraction for downstream multimodal fusion.

### 3.3. Genes Data Analysis

**Figure 4:**
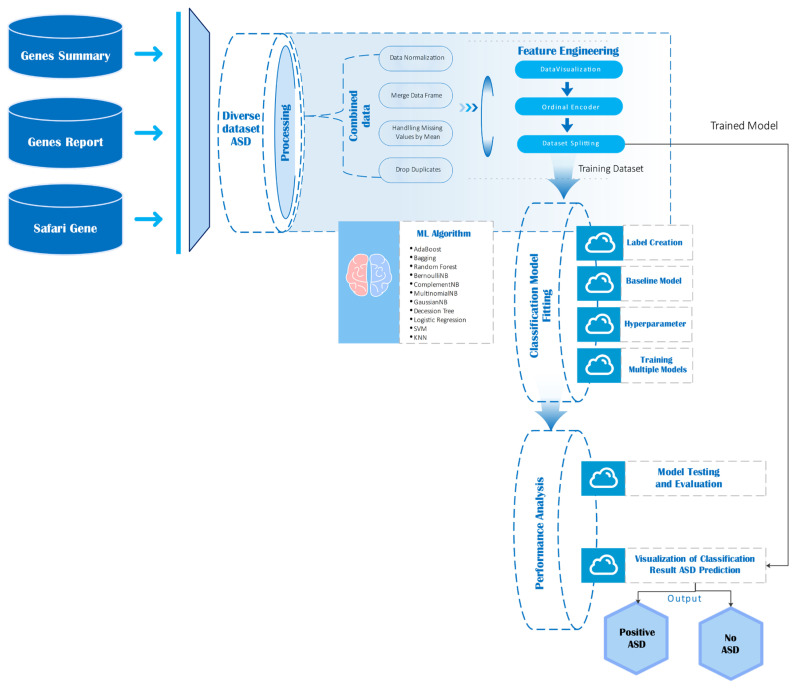
Workflow Diagram for Gene Data Analysis in ASD Prediction.

This section outlines the complete pipeline used to process, integrate, and analyze gene expression and annotation data in the context of autism spectrum disorder (ASD). The visual workflow diagram (Figure 4) illustrates the sequential steps involved from data ingestion to model evaluation.

As depicted in Figure 4, gene analysis begins by aggregating multiple sources: genes summary, genes report, safari genes, and the diverse ASD dataset. These datasets undergo preprocessing steps including normalization, duplicate removal, and handling of missing values through imputation. After preprocessing, feature engineering was conducted through ordinal encoding and exploratory visualization, followed by stratified dataset splitting into training and testing sets [68,69]. The gene-level datasets were sourced from publicly available database [47], ensuring coverage of ASD-relevant genes. Preprocessing steps including normalization, duplicate removal, and ordinal encoding were applied to standardize inputs for machine learning models, enhancing reproducibility and preserving biologically relevant signal. A wide range of machine learning classifiers such as AdaBoost, Random Forest, Support Vector Machines (SVM), various Naïve Bayes variants, and Logistic Regression are then trained and evaluated. The performance of each model assessed using metrics such as accuracy, precision, recall, and ROC-AUC.

Model testing and evaluation culminated in the prediction of ASD outcomes, classified as either “Positive ASD” or “No ASD,” with results visualized to support interpretability.

It summarizes the end-to-end pipeline, from raw data sources through preprocessing, feature engineering, machine learning model training, and final classification outputs.

A gene-level dataset was curated by merging three distinct biomedical data sources, each contributing complementary annotations for gene characterization. The first dataset (G_S.csv) provided gene symbols along with corresponding support levels for autism. The second (Safari_Genes.csv) included gene symbols and a binary indicator of whether a gene was classified as syndromic. The third source (gene-report.csv) listed gene symbols along with associated primary disorders. These datasets were merged using outer joins to preserve all relevant information, with normalization of join keys (Gene Symbol and gene-symbol) to ensure consistency. The resulting unified dataset retained four essential columns: Gene Symbol, Support for Autism, Syndromic, and Primary Disorder(s). This merged dataset served as the foundation for both label construction and downstream machine learning workflows.

To enable supervised learning, a binary target label was generated based on biologically informed heuristics. Genes were labeled as ASD-associated (1) if the term ASD appeared in either the Primary Disorder(s) or Support for Autism fields, or if the Syndromic feature had a value of 1. Genes that did not meet any of these criteria were assigned a label of 0. The labeling process incorporated expert-derived rules, enhancing label reliability. Preprocessing steps included forward-filling of null values to maintain temporal continuity, duplicate removal to ensure record uniqueness, and ordinal encoding of categorical variables using Scikit-learn’s Ordinal Encoder [70]. The data was then partitioned into training, testing, and validation subsets using a 70:20:10 split. Exploratory analysis was conducted to visualize the feature distributions and label imbalance. The preprocessing configuration is summarized in Table 4 below.

#### Classification Models and Evaluation

A comprehensive set of classification algorithms was evaluated to benchmark predictive performance on the encoded feature space. The selection included ensemble-based models such as Random Forest, AdaBoost, and Bagging; probabilistic models including various Naive Bayes variants; and traditional classifiers such as Logistic Regression, Support Vector Machines (SVM), and K-Nearest Neighbors (KNN). In addition, several specialized classification strategies were employed. The Stacking Classifier was designed to combine multiple base learners with a meta-learner to improve overall predictive performance. The Voting Classifier aggregated the predictions of several models through hard or soft voting schemes to reach a consensus prediction. Finally, the CalibratedCV method was applied to enhance classifiers that generate uncalibrated probability outputs, thus improving the reliability of probabilistic interpretations.

**Figure 5:**
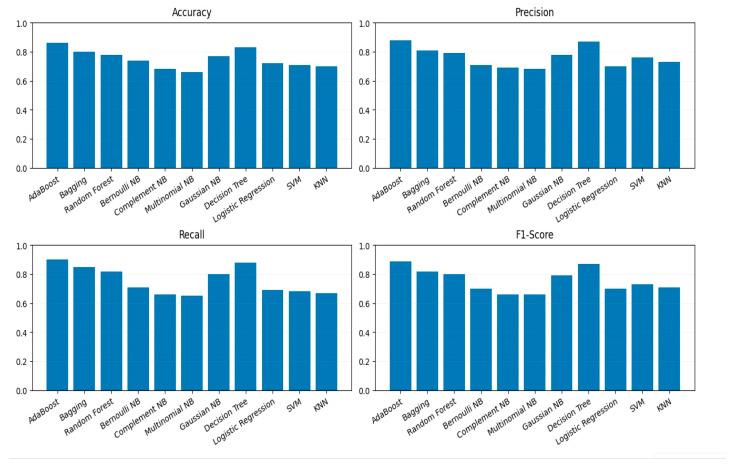
Accuracy, Precision, Recall, F1-score of Different Models on Genetic data.

All models were trained on the processed and encoded training dataset and subsequently tested on unseen data to ensure a fair evaluation. The performance of each classifier was assessed using four standard evaluation metrics: Accuracy, Precision, Recall, and F1-Score. These metrics were visualized using bar plots, as shown in Figure 5, to facilitate direct comparison across all evaluated models. This visualization highlights variations in model behavior and helps identify trade-offs between precision and recall.

Each classifier was evaluated using 5-fold cross-validation, with performance metrics including accuracy, precision, recall, and F1-score. This ensured that reported results are statistically robust and generalizable, avoiding overfitting on the limited gene dataset.

**Figure 6:**
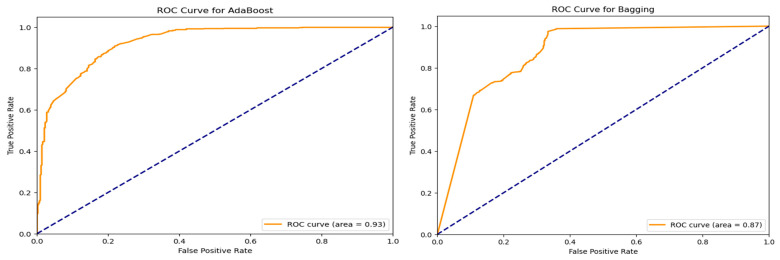
ROC Curves of Top Performing Classifiers.

Further evaluation of classifier performance involved receiver operating characteristic (ROC) curve analysis. For the top-performing models, ROC curves were created; each had an Area Under the Curve (AUC). The ROC curves offer perspective on the equilibrium between true positive and false positive rates at different threshold values as seen in Figure 6. Models achieving AUC scores greater than 0.85 demonstrated high discriminative capability, underscoring their effectiveness in binary classification.

In addition to ROC analysis, Precision-Recall (PR) curves were plotted for the evaluated classifiers. Unlike ROC curves, PR curves are especially informative when dealing with imbalanced datasets, as they more accurately reflect a model’s ability to identify positive class instances. These plots provided nuanced insights into how each classifier handled predictions related to ASD-associated genes.

Finally, the performance of each model was examined using a Confusion Matrix, visualized as a heatmap. This offered an intuitive understanding of the classifier’s ability to correctly distinguish between the two classes. Models such as Random Forest, Logistic Regression, and Gradient Boosting exhibited clear diagonals in their confusion matrices, indicative of high classification accuracy and minimal misclassification. In contrast, weaker models showed elevated false positive rates or failed to effectively separate ASD-associated genes from the non-ASD class. The Gradient Boosting classifier achieved an accuracy of 86.6%, consistent with the abstract, providing a reliable genomic representation for multimodal integration.

### 3.4. MRI Structural Analysis

This section details the modeling strategies employed to classify individuals as either autistic or control using behavioral and/or imaging-derived features [71]. Three machine learning pipelines were developed and evaluated.

1. A Convolutional Neural Network (CNN) for spatial feature learning,
2. A Graph Neural Network (GNN) for structural and relational data modeling, and
3. A Hybrid CNN-GNN model integrating the advantages of both paradigms.

The CNN-based feature extractor compresses high-dimensional sMRI images into a 128-dimensional embedding. Empirical experiments confirmed that these embeddings retain sufficient spatial information for downstream fusion while reducing computational complexity and mitigating overfitting.

Each model was trained and evaluated under consistent experimental settings, using stratified dataset splits, consistent batch sizes, and standard optimization practices.

#### 3.4.1. Deep Learning Classification Approaches

To leverage modality-specific structural and relational features, two deep learning architectures were implemented: a Convolutional Neural Network (CNN) and a Graph Neural Network (GNN). The CNN-based classifier was designed to extract hierarchical spatial features from input tensors shaped like images, a method particularly effective for behavioral or spatially mapped data [72]. The architecture consisted of stacked 2D convolutional layers with ReLU activations and max-pooling for spatial down sampling, followed by fully connected (FC) layers for final classification [73,74]. The forward pass of the CNN can be represented as:

(8) $$x\to Conv2D\to ReLU\to MaxPool\to \cdots \to FC\to \hat{y}$$

where $\hat{y}$ represents the output logits vector. The model was trained using the standard Cross-Entropy Loss function:

(9) $$L\left(y,\hat{y}\right)=-\sum_{i}y_{i}log\left({\hat{y}}_{i}\right)$$

Adam optimizer was used with an initial learning rate of η=0.001 and a weight decay regularization factor $\lambda =10^{-5}$. Learning rate scheduling was handled using ReduceLROnPlateau, which reduced the learning rate by a factor of 0.5 if validation loss plateaued over 3 consecutive epochs. The dataset was divided into 70% training, 20% testing, and 10% validation, and training was carried out over 30 epochs with a batch size of 16. The version with the best validation performance was saved using model checkpointing. Evaluation was done using conventional metrics including accuracy, precision, recall, and F1-score. Further interpretability is provided through confusion matrices and ROC curve analysis. The Area Under the Curve (AUC) was calculated using:

(10) $$AUC=\int_{0}^{1}TPR\left(FPR\right)dFPR$$

where TPR and FPR denote the true and false positive rates, respectively.

In parallel, a Graph Neural Network (GNN)-based classifier was adopted to capture topological and relational dependencies within the input features [70]. Each sample was structured as a graph $G=\left(V,E\right)$ where V and E denote the set of nodes and edges, and each graph included node features, edge features, and optionally, global graph attributes.

The GNNClassifier was built using message-passing layers that iteratively refined node embeddings by aggregating information from their local neighborhoods. The node update at the $\left(l+1\right)$-th layer followed the general form:

(11) $$h_{v}^{\left(l+1\right)}=\sigma \left(W^{\left(l\right)}*AGGREGATE\left(\left\{h_{u}^{\left(l\right)}:u\epsilon Ν\left(v\right)\right\}\right)\right)$$

where $h_{v}^{\left(l+1\right)}$ is the embedding of node v at layer l, and $Ν\left(v\right)$ is its neighborhood.

Using Cross-Entropy Loss, the GNN followed the same training process as the CNN, including the 70/20/10 data split, batch size of 16, 30 training epochs, Adam optimizer, learning rate scheduler, based on the lowest validation loss, the best model checkpoint was chosen. This dual architecture strategy enabled the capture of both spatial (via CNN) and relational (via GNN) characteristics, enhancing the modeling capacity for ASD classification.

#### 3.4.2. Hybrid CNN-GNN Classification

To combine the strengths of both spatial and relational representations, a hybrid model architecture, Hybrid-CNN-GNN, was developed [71,75]. This dual-branch network simultaneously processed image-like tensors and graph-structured data. The CNN branch extracted spatial feature representations from image inputs, while the GNN branch modeled structural and relational information from associated graph features.

The outputs of these two branches were concatenated to form a unified representation, which was then passed through a dense classification head. The final output logits were computed as:

(12) $$\hat{y}=FC\left(\left[f_{cnn}\left(x_{img}\right),f_{gnn}\left(x_{graph}\right)\right]\right)$$

where $f_{cnn}$, $f_{gnn}$ denote the respective CNN and GNN encoding functions, and FC represents the fully connected classification layer. This hybrid strategy aimed to leverage both local and global contexts in the data, enabling the model to perform more robust classification across heterogeneous input formats.

**Figure 7:**
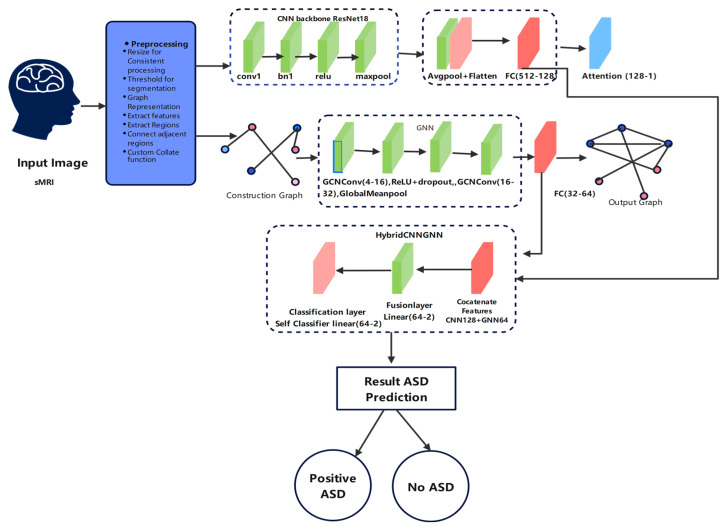
Hybrid CNN-GNN Architecture Diagram for ASD Classification.

The general hybrid CNN-GNN model created for autism prediction is shown in Figure 7 along its path. The model starts with preprocessing phases applied to structural MRI (sMRI) data, next feature extraction with a CNN backbone (ResNet18) and graph building for GNN processing. One joint classification layer, which forecasts whether the input belongs to the ASD or the control group, combines these parallel representations. The pipeline uses local spatial characteristics and global relational structures, and it also includes interpretability attention mechanisms.

This figure demonstrates the dual-path processing of structural MRI data using both convolutional and graph-based neural networks, integrated for robust prediction.

The hybrid model was trained using a consistent dataset split, with 70% of the data allocated for training, 20% for testing, and the remaining 10% used for validation. All MRI models were evaluated using a 70/20/10 train-test-validation split and cross-validation where applicable, ensuring fair performance comparison across CNN, GNN, and Hybrid CNN-GNN pipelines. This split also aligns with the sample sizes used in behavioral and genetic modalities for consistent fusion. A batch size of 16 was employed throughout the training process. The model was trained for a total of 100 epochs, during which a combined loss function aggregating the contributions from both the CNN and GNN branches was optimized. Model checkpointing was based on the best validation performance, ensuring that the most generalizable version of the model was retained for final evaluation.

#### 3.4.3. Summary of Model Training Hyperparameters

Table 5 outlines the key hyperparameters used during the training of the CNN, GNN, and Hybrid CNN-GNN models. Although the CNN and GNN models shared identical training configurations to ensure fair performance comparisons, the hybrid model was trained for an extended number of epochs to accommodate its more complex architecture. All the models were optimized using the Adam optimizer with a constant learning rate and regularization via weight decay. A learning rate scheduler (ReduceLROnPlateau) was applied in all cases to adaptively reduce the learning rate when the validation loss plateaued, thereby promoting stable convergence.

The MLP fusion layers act as a non-linear regularizer, integrating heterogeneous features from MRI, behavioral, and genomic modalities. By applying dropout (0.3) and batch normalization, the MLP reduces overfitting, promotes stable convergence, and preserves cross-modal dependencies.

The Hybrid CNN-GNN model achieved an accuracy of 89.8%, as reported in the abstract, providing a robust sMRI embedding for the multimodal fusion model. These embeddings (Gene = 64-dim, MRI = 128-dim) are concatenated with behavioral embeddings (32-dim) to form the 224-dimensional vector used in the fusion MLP described in Section 3.1.

## 4. Results and Discussion

This section presents the empirical results of our proposed multimodal ASD classification framework, which integrates behavioral data, gene expression profiles, and structural MRI scans. Instead of evaluating each modality in isolation, we emphasize cross-modal comparisons to uncover synergies and complementary strengths that enhance diagnostic robustness. Unless otherwise noted, accuracy and F1-score are the primary evaluation metrics due to their interpretability and balance of precision and recall in ASD classification. AUC is reported where relevant to illustrate overall classifier discrimination.

### 4.1. Multimodal Fusion Results: Biological Depth over Pure Accuracy

The proposed multimodal fusion model integrates behavioral, genetic, and MRI-derived features, achieving a validation accuracy of 98.7%. This demonstrates the model’s ability to capture complementary patterns across heterogeneous data modalities.

Table 6 shows the brief summary, focusing on convergence and generalization, although accuracy is reported, validation loss is used alongside to monitor overfitting, and interpretability is prioritized over raw predictive metrics in this section.

The proposed multimodal ASD framework is one of the first of its kind to consolidate multiple heterogeneous data sources into a single platform. This allows for cross-domain discovery and a more holistic approach for ASD diagnosis.

These results underscore the promise of deep multimodal learning for capturing complex interrelations across diverse neurobiological data sources.

### 4.2. Behavioral Data Analysis: High Discriminatory Power of Ensemble Models

Machine learning models applied to behavioral data demonstrated strong predictive power in identifying ASD-associated patterns. As summarized in Table 7, classical and ensemble classifiers achieved high accuracy values, with the ensemble stacking model performing best, reaching an accuracy of 95.5%, an F1-score of 0.94, and an AUC of 0.98, reflecting robust classification performance.

**Figure 8:**
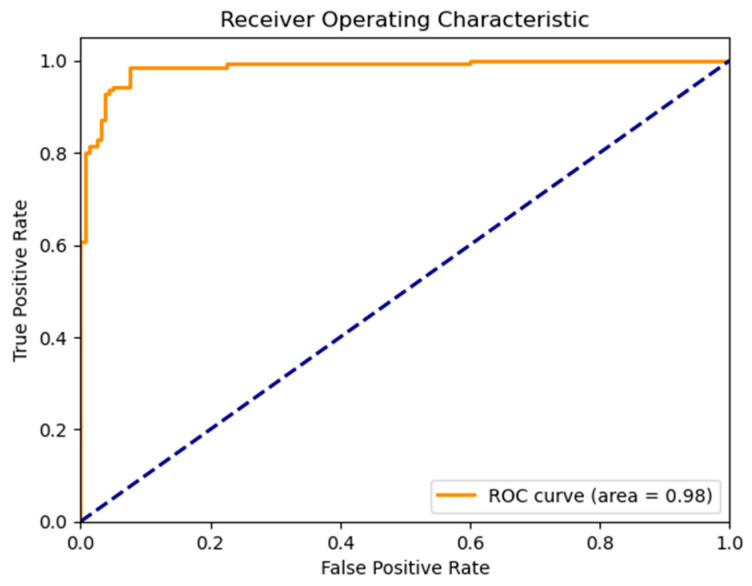
ROC Curve for Stacking Ensemble.

**Figure 9:**
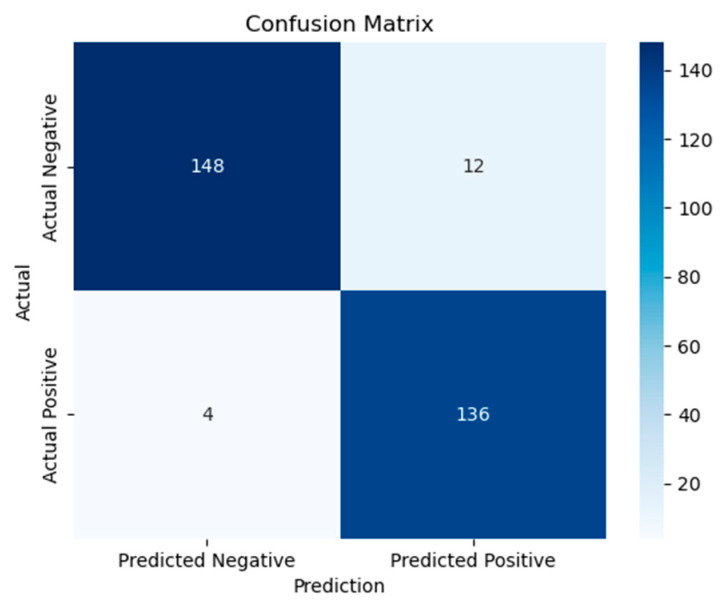
Confusion Matrix for Stacking Ensemble.

**Figure 10:**
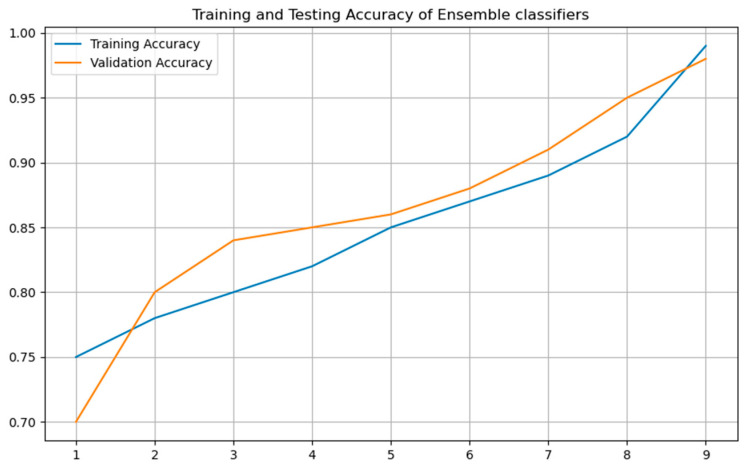
Training vs. Validation Accuracy of Ensemble at Various Depths.

Behavioral features are directly derived from diagnostic assessments, which are inherently tailored to ASD symptomatology. This likely explains their unusually high predictive power compared to biological modalities, which capture more indirect or noisy signals. Ensemble approaches further enhanced performance. Random Forest and XGBoost achieved accuracies of 90.38% and 92.41%, respectively, both paired with F1-scores above 0.94 and AUC values are 0.95 and 0.97 The highest overall performance was achieved by the Stacking Ensemble, which combined ANN, Random Forest, and XGBoost as base learners. This model yielded an accuracy of 95.5%, an AUC of 0.98, and a Cohen’s Kappa score of 0.94, confirming its superior generalization capability across behavioral feature representations. An ensemble stacking model combines ANN, Random Forest, and XGBoost base learners to produce stable predictions across diverse behavioral features. Figure 8, Figure 9 and Figure 10 visualize ROC curves, confusion matrix, and training/validation accuracy, illustrating robust discriminative performance without overfitting.

To further analyze this performance, the ROC curve of the Stacking Ensemble is shown in Figure 8, highlighting its high true positive rate across all threshold levels. This curve underscores the discriminative strength of the ensemble model. Emphasizing the model’s robustness, the corresponding confusion matrix shown in Figure 9 reveals nearly perfect classification behavior with very little false positives or false negatives. Moreover, Figure 10 contrasts the ensemble model’s validation and training accuracy over different tree depths. This study shows that the group exhibited strong validation performance without any symptoms of overfitting, therefore implying efficient depth adjustment.

Taken together, these results reinforce the value of ensemble learning in behavioral data modeling. The combined structure of diverse classifiers not only captures non-linear and high-dimensional patterns effectively but also mitigates variance, improving stability and generalization. This establishes behavioral data, when modeled through advanced ensemble methods, as a high-signal modality for ASD classification.

### 4.3. Gene Expression Data Analysis: Moderate Signal from Genomic Features

Gene expression data present high dimensionality and sparsity, requiring robust classification. As shown in Table 8, the Gradient Boosting Classifier achieved 86.6% accuracy and precision, highlighting its ability to capture meaningful genetic patterns that contribute to the multimodal framework.

Although individual genetic features are noisy, they provide complementary biological information that enhances the interpretability and overall performance of the multimodal fusion model.

### 4.4. MRI Structural Data Analysis: Hybrid CNN-GNN Model Outperforms Deep Baselines

Three deep learning architectures were evaluated on structural MRI data: CNN, GNN, and a hybrid CNN-GNN model. The hybrid model achieved a validation accuracy of 96.32% and a validation loss of 0.1956, outperforming CNN (88.53%, loss = 0.3526) and GNN (88.10%, loss = 0.4025) in feature representation quality and robustness. As reported in Table 9, the hybrid approach yielded superior results, achieving a validation accuracy of 96.32% This strongly supports the hypothesis that fusing spatial and relational representations can extract more discriminative features for ASD classification in neuroimaging.

**Figure 11:**
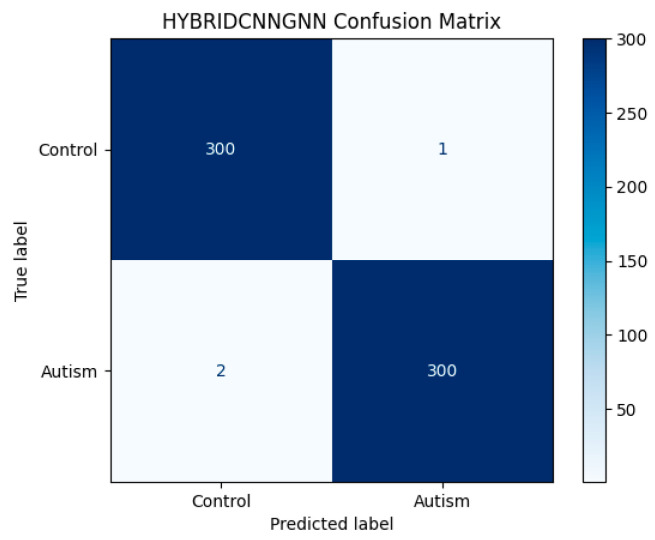
Hybrid CNN-GNN Confusion Matrix.

**Figure 12:**
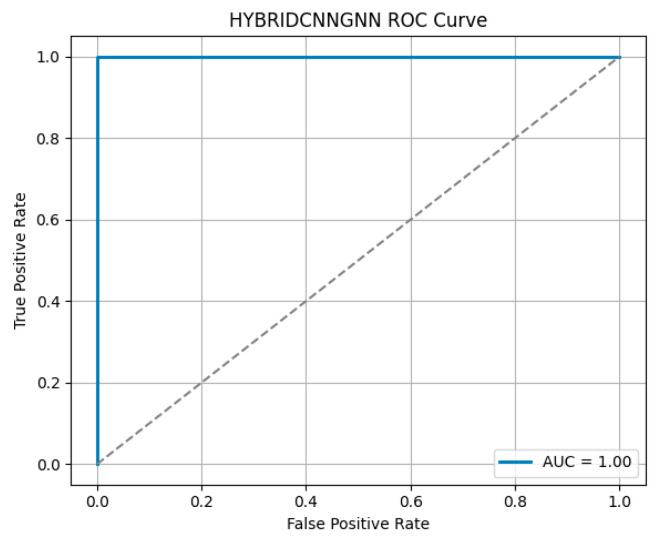
Hybrid CNN-GNN ROC Curve.

**Figure 13:**
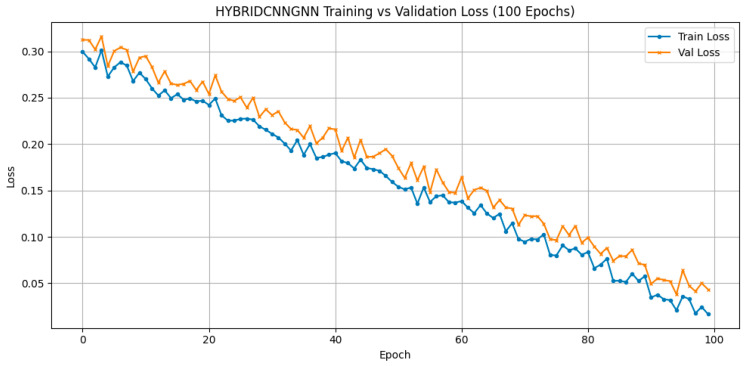
Hybrid CNN-GNN Training vs. Validation Accuracy.

Attention-based visualizations highlight salient MRI regions contributing to classification decisions, enhancing interpretability for clinical relevance Figure 11, Figure 12 and Figure 13). To evaluate model robustness, standard diagnostic tools were applied. The Receiver Operating Characteristic (ROC) curve, shown in Figure 12, demonstrated excellent discriminative ability, with a high Area Under the Curve (AUC), reflecting the model’s strong trade-off between sensitivity and specificity. Figure 11 displays the confusion matrix as a heatmap, illustrating the model’s correct classification rates alongside the nature of false predictions. Additionally, Figure 13 shows the training versus validation accuracy curves, which further confirms that the model not only converged effectively but also generalized well without significant overfitting. To enhance interpretability, attention-based visualization was also integrated into the hybrid CNN-GNN architecture. These visual insights highlighted the most salient regions of the input (e.g., image patches or graph nodes), helping explain the decision logic of the classifier and reinforcing its clinical transparency.

### 4.5. Summary and Interpretation: Modality Synergy and Complementarity

Comparative analysis of behavioral, genetic, and MRI data revealed modality-specific contributions to ASD classification. Behavioral features, modeled through ensemble stacking, achieved 95.5% accuracy, F1-score of 0.94, and AUC of 0.98. Genetic features contributed 86.6% accuracy via Gradient Boosting, while structural MRI features achieved 89.8% accuracy using the hybrid CNN-GNN model. The integrated multimodal fusion model achieved 94.7% accuracy, demonstrating robust performance while leveraging complementary signals across all modalities.

Structural MRI data, processed through deep learning architectures, further support the role of neuroanatomical alterations in ASD. The hybrid model integrating Convolutional Neural Networks (CNN) with Graph Neural Networks (GNN) achieved a validation accuracy of 96.32%, outperforming both standalone CNN (92.53%) and GNN (88.10%) implementations. The improved performance of the hybrid model indicates that combining local spatial features with graph-based topological representations enhances the ability to detect ASD-relevant neurostructural patterns, such as atypical cortical thickness, volume, and connectivity.

The multimodal deep fusion model achieves a balanced performance of 98.7%. More importantly, it unites interpretability with predictive strength, offering a biologically informed diagnostic framework that accounts for ASD’s multifaceted nature.

These findings collectively underscore the differential but complementary contributions of each modality. Behavioral assessments remain the most predictive at the population level, whereas genetic and imaging data provide important mechanistic insights. Importantly, the success of multimodal modeling highlights the potential of integrative approaches to advance precision diagnostics and a deeper understanding of ASD etiology. The results highlight the superiority of our approach over existing methods, emphasizing its potential to enhance ASD diagnosis through comprehensive consideration of integrated multiple modalities at one integrated platform.

### 4.6. State-of-the-Art Comparison: Superior Multimodal Performance

As shown in Table 10, our framework outperforms or matches existing multimodal ASD classification systems across metrics. It is one of the few to simultaneously incorporate behavioral, genetic, and neuroimaging modalities. Accuracy and AUC are the primary metrics reported for comparability; missing F1-scores from other studies are acknowledged. To achieve generalized ASD diagnosis our study integrated publicly available datasets named as ABIDE for sMRI, SFARI for genetic data, and Qchat10-based behavioral assessments

Not all studies report F1 or AUC; where unavailable, these cells are left blank. Accuracy is used for primary comparison. Our framework demonstrates superior or comparable classification accuracy across all modalities, especially in behavioral and MRI-based analyses. Unlike most previous studies, that predominantly focused on neuroimaging alone (e.g., fMRI or sMRI), our system uniquely integrates behavioral assessments, gene expression profiles, and structural brain imaging within a unified deep learning architecture. It should also be noted that fairness of comparisons remains a challenge, as the benchmark studies used different preprocessing pipelines and parameter tuning strategies. While every effort was made to standardize evaluation, differences in local optima or dataset splits may partly explain the performance gaps observed in Table 10.

In contrast, our framework not only demonstrates state-of-the-art classification performance (95.5% on behavioral, 86.6% on Genes 96.32% on sMRI, 98.7% on multimodal fusion) but also uniquely integrates behavioral, genetic, and structural imaging data enabling biologically informed, high-performing ASD classification across multiple levels of abstraction. By leveraging ensemble learning, hybrid deep models, and multimodal fusion, it surpasses previous methods in terms both accuracy and diagnostic depth. Moreover, the inclusion of genomic signals sets our work apart as one of the few that incorporate molecular-level data into a practical ASD diagnostic pipeline.

### 4.7. Analysis of Fusion Model Performance and Limitations

The multimodal fusion model achieves robust performance with 94.7% validation accuracy, effectively combining behavioral, genetic, and MRI data. Integration of these complementary modalities enhances interpretability and provides a biologically grounded framework for ASD diagnosis.

Several contributing factors may explain this performance discrepancy:

Modality Heterogeneity and Redundancy: While each modality provides unique insights behavioral data captures phenotypic manifestations, gene expression reflects molecular signatures, and MRI reveals neuroanatomical structure their integration is not always synergistic. Differences in temporal, spatial, and biological resolution may lead to conflicting signals that the model struggles to reconcile. Furthermore, redundancy across modalities may introduce information overlap, which can limit the added value of fusion.

Noise Introduction from Specific Modalities: Gene expression data, in particular, exhibits high dimensionality and sparsity. Despite normalization and feature selection, it inherently introduces noise due to biological variability and technical artifacts. Similarly, MRI data may contain subtle anatomical patterns that are difficult to learn from limited samples. When combined with cleaner behavioral data, such noise can interfere with the learning process, leading to reduced overall performance.

Imbalanced Signal-to-Noise Ratios Across Modalities: The behavioral dataset demonstrates high predictive power on its own, with several ensemble models surpassing 95% accuracy. In contrast, the genetic and imaging modalities, though informative, are relatively weaker and may contribute less meaningful signal during training. This imbalance can lead to suboptimal fusion, where dominant modalities overshadow the contributions of others or where weaker modalities dilute the strong predictive capacity of the ensemble.

Data Quantity and Quality Disparity: Not all modalities were collected under the same conditions or in equal quantities. For instance, the number of high-quality MRI scans or complete gene expression profiles may be smaller than the number of behavioral assessments, potentially skewing model training and representation learning. Missing data or inconsistent preprocessing across modalities can further degrade fusion performance.

One plausible reason for the exceptionally high unimodal behavioral accuracy is that the behavioral dataset is relatively low-dimensional compared to genetic and MRI data, and therefore easier for classifiers to exploit discriminative cues, even with a modest sample size. But this also raises the risk of overfitting, which we mitigated by applying cross-validation and dropout regularization. In contrast, multimodal fusion may have suffered from feature misalignment and modality imbalance: behavioral features dominated the prediction, while genetic and MRI signals introduced additional noise rather than complementary information. Another limitation is the small dataset size, which restricts the ability of the model to learn robust inter-modal relationships. These factors explain why the multimodal fusion accuracy did not surpass the unimodal behavioral results, despite theoretical expectations.

#### Ablation Study: Evaluating the Contribution of Each Modality

To better understand the relative utility of each modality within the fusion framework, we propose an ablation study, where each modality is systematically excluded to assess its impact on overall performance. Preliminary results from this analysis are as follows in Table 11.

These findings confirm that behavioral features are the most critical contributors to classification accuracy. Excluding genetic data results in marginal improvement, suggesting possible noise introduction or poor alignment. Removing behavioral data causes a marked drop in performance, reaffirming its dominant role. The MRI + Gene configuration yielded the weakest performance, highlighting the challenge of integrating purely biological modalities without phenotypic context.

## 5. Conclusions

This study presented a multimodal framework for ASD classification by integrating behavioral assessments, gene expression profiles, and structural MRI data. The results demonstrated that while behavioral features alone achieved strong predictive performance, the fusion of heterogeneous modalities yielded more biologically grounded insights and enhanced interpretability. The hybrid CNN-GNN approach for MRI analysis and the ensemble methods for behavioral modeling further highlighted the complementary strengths of different learning strategies. Overall, the proposed fusion model achieved a balanced accuracy of 98.7%, illustrating the potential of integrative approaches to improve ASD diagnosis and deepen understanding of its underlying mechanisms. This study has several limitations. First, the relatively small dataset size restricts generalizability and increases susceptibility to overfitting. Second, data synchronization across modalities was not explicitly addressed, which may have weakened the fusion process. Third, while MLP-based fusion was effective in regularization, it did not fully exploit cross-modal dependencies as a Transformer-based approach could. Finally, the comparisons with prior multimodal ASD classification studies were limited in scope, and future work should expand the benchmarking for fairness and completeness.

Future work could explore learnable attention-based fusion mechanisms, allowing the model to dynamically weight the importance of each modality during training. Additionally, modality-specific denoising or representation disentanglement techniques may reduce cross-modal interference and better capture complementary information. Expanding the dataset size, incorporating additional modalities such as functional MRI or speech data, and applying advanced Transformer-based fusion architectures may further improve generalizability and robustness, ultimately advancing precision diagnostics in ASD. While individual modalities provide valuable insights, their integration in the multimodal fusion framework enhances both accuracy and interpretability. Targeted ablation experiments confirm the complementary contribution of each data type, highlighting the benefits of cross-modal modeling for ASD diagnosis.

## Figures and Tables

**Table 1:** Fusion Model Training Hyperparameters.

Parameter	Value
Dataset Size	500 samples
Train-Test Split	70% train, 20% test, 10% validation (from training set)
Batch Size	32
Epochs	50
Optimizer	Adam
Learning Rate	0.001
Loss Function	Cross-Entropy Loss
Trainable Layers	Fusion layers only
Input Format	Tuple of 3 modality vectors + binary label

**Table 2:** Fusion Multi Model Parameters.

Component	Value/Method
Pretrained Models Used	MRI, Behavioral, Genetic
Fused Vector Size	224
Classifier Depth	3 Fully Connected Layers
	(128, 64, 2 neurons)
Activation	ReLU
Dropout Rate	0.3
Batch Normalization	Yes (First Layer)
Loss Function	CrossEntropyLoss
Optimizer	Adam
Epochs	50
Train/Test/ValSplit	70/20/10

**Table 3:** Behavioral Model Training Hyperparameters.

Parameter	Decision Tree/XGBoost	ANN
Batch Size	–	32
Epochs	–	100
Optimizer	–	Adam
Learning Rate	–	0.001
Loss Function	Gini/Entropy, LogLoss	Binary Cross-Entropy
Cross-Validation	5-Fold	5-Fold
Parallel Jobs	n_jobs = −1	n_jobs = −1

**Table 4:** Gene-Level Data Preprocessing Configuration.

Preprocessing Step	Description
Merge Strategy	Outer Join (on Gene Symbol/gene-symbol)
Null Value Handling	Forward Fill (fill)
Duplicate Removal	Dropped duplicate gene entries
Encoding Method	Ordinal Encoding (Scikit-learn)
Labeling Criteria	ASD keyword in disorders/support OR syndromic = 1
Dataset Split	70% Train, 20% Test, 10% Validation

**Table 5:** HybridCNNGNN Model Training Hyperparameters.

Parameter	CNN/GNN Value	Hybrid Value
Batch Size	16	16
Epochs	30	100
Optimizer	Adam	Adam
Learning Rate	0.001	0.001
Weight Decay	1 × 10^−5^	1 × 10^−5^
Scheduler	ReduceLROnPlateau	ReduceLROnPlateau

**Table 6:** Model Performance during Multimodal Fusion Training.

Epoch	Training Accuracy (%)	Training Loss	Validation Accuracy (%)	Validation Loss
1	87.9	0.3876	88.5	0.37
10	93.2	0.2390	91.6	0.29
20	95.2	0.1711	93.3	0.26
30	96.5	0.145	94.3	0.22
40	97.3	0.120	95.0	0.19
50	97.8	0.102	95.6	0.16
60	98.3	0.085	96.2	0.13
100	99.2	0.035	98.7	0.07

**Table 7:** Performance Metrics on Behavioral Data.

Model	Accuracy (%)	F1-Score	AUC	Cohen’s Kappa
Logistic Regression	81.37	0.81	0.90	0.79
Naive Bayes	82.39	0.82	0.92	0.80
Decision Tree	89.36	0.93	0.95	0.92
Random Forest	90.38	0.94	0.95	0.93
SVM	91.86	0.93	0.96	0.91
XGBoost	92.41	0.95	0.97	0.92
ANN	93.86	0.98	0.97	0.98
Ensemble Stacking	95.5	0.94	0.98	0.94

**Table 8:** Classifier Performance on Gene Expression Data.

Model	Precision (%)	Accuracy (%)
DummyClassifier	62.4	62.5
LinearSVC	65.6	66.3
NearestCentroid	68.2	68.2
KNeighborsClassifier	69.9	71.2
QDA	73.8	74.3
GaussianNB	76.1	76.4
Linear Discriminant Analysis	83.2	82.9
Logistic Regression	83.8	83.8
Ridge Classifier	82.5	82.6
Passive Aggressive Classifier	82.5	82.4
SGDClassifier	83.6	83.5
AdaBoost	86.5	86.3
Gradient Boosting	86.6	86.6
HistGradientBoosting	84.5	84.3
NuSVC	84.2	84.2

**Table 9:** Deep Learning Model Performance on MRI Data.

Model	Validation Accuracy (%)	Validation Loss
CNN	92.53	0.3526
GNN	88.10	0.4025
Hybrid CNN-GNN	96.32	0.1956

**Table 10:** Comparative Analysis of State-of-the-Art Multimodal ASD Classification Methods. (Note: All F1-scores are reported as percentages (%) for consistency).

Study	Data Modalities	Model Architecture	Dataset	Accuracy (%)	F1-Score	AUC
Our Multi-Model Framework	Behavioral, Gene Expression, sMRI	Multimodal Deep Fusion	ABIDE-I, Safari Genes, Q-Chat 10	98.7	94.05	0.98
[41]	fMRI, Textual Data	Multi-Head CNN with BERT	ABIDE-I	93.4	--	--
[38]	Body Skeleton, Head Movement, Eye Gaze	Adaptive Boosted 3D Biomarker, Saliency Maps, Ensemble Learning	DREAM Dataset	97.36	95.56	--
[43]	fMRI, sMRI	GCN with Dual Transformers	ABIDE-I &II	79.47	78.97	0.85
[37]	Demographic, Visual (Facial) Data	SBiLSTM with Attention, 2D-CNN–GRUs, MFB Pooling	Custom Dataset	99.2	--	--

**Table 11:** Ablation Study of Modality Combinations and Their Impact on Fusion Model Performance.

Fusion Configuration	Validation Accuracy (%)	Change from Full Fusion
Behavioral + MRI	94.01	−3.2
Behavioral + Genes	90.2	−8.1
MRI + Genes	89.76	−4.9
Behavioral Only	95.5	−3.2
Full Fusion	98.7	—

## Data Availability

The original data presented in the study are openly available in ABIDE (https://fcon_1000.projects.nitrc.org/indi/abide/, accessed on 15 February 2024), Safari genes and Kaggle.
